# Solving the Border Control Problem: Evidence of Enhanced Face Matching in Individuals with Extraordinary Face Recognition Skills

**DOI:** 10.1371/journal.pone.0148148

**Published:** 2016-02-01

**Authors:** Anna Katarzyna Bobak, Andrew James Dowsett, Sarah Bate

**Affiliations:** 1 Psychology Research Centre, Faculty of Science and Technology, Bournemouth University, Poole, Dorset, United Kingdom; 2 School of Psychology, University of Aberdeen, Aberdeen, United Kingdom; Technion Israel Institute of Technology, ISRAEL

## Abstract

Photographic identity documents (IDs) are commonly used despite clear evidence that unfamiliar face matching is a difficult and error-prone task. The current study set out to examine the performance of seven individuals with extraordinary face recognition memory, so called “super recognisers” (SRs), on two face matching tasks resembling border control identity checks. In Experiment 1, the SRs as a group outperformed control participants on the “Glasgow Face Matching Test”, and some case-by-case comparisons also reached significance. In Experiment 2, a perceptually difficult face matching task was used: the “Models Face Matching Test”. Once again, SRs outperformed controls both on group and mostly in case-by-case analyses. These findings suggest that SRs are considerably better at face matching than typical perceivers, and would make proficient personnel for border control agencies.

## Introduction

National security relies on accurate ID-to-person comparison. Passport checks are now often performed by electronic gates with specialised image processing software, yet when this method fails, or, within the European Union, when an individual is from outside of the European Economic Area, the ID bearer is sent directly to a passport officer. Thus, the identity decision ultimately relies on the ID-to-person comparison skills of a human observer. Although this image-to-person comparison is the most commonly used means of assessing one’s identity without automated technology (e.g. fingerprint or iris analysis), a large body of literature indicates that there are large individual differences in unfamiliar face recognition within the typical population [1–3]. This variability in performance can at least partly be attributed to the difficulty of the task, and even face matching is prone to errors even though no demands are placed on memory [1,3,4]. Unfamiliar face matching is particularly sensitive to changes in the device used to take images [4], viewpoint [4], lighting [5] and the passage of time between image capture [6].

These decrements in performance can be explained within Bruce and Young’s [7] modular and sequential model of face recognition. Specifically, the model posits that in the initial encoding stage, faces are stored using pictorial codes. As such, when individuals are presented with the same images at study and test, the identity discriminations are fast and highly accurate [8]. This pattern of findings is not replicated, however, when two images differ in viewpoint, lighting or the device used to capture them. This decrease in performance is thought to reflect a disruption in the pictorial codes used to store the identities [1,4]. With gradual exposure to various instances of the same face, structural codes produce a more stable view-independent representation that is easily recognisable under various conditions. Pertinently, a recent study by Dowsett and Burton [9] suggests that recognition accuracy can be improved by increasing the variability of information available at encoding, by presenting multiple images of the same person. However, the brevity of security checks on national frontiers does not allow for such stable structural representations to be formed, and it is thus important to find alternative ways of safeguarding accuracy during identity document (ID) to-person comparisons.

In support of Bruce and Young’s [7] model, numerous studies have reported large individual differences in face matching performance in the typical population. For instance, Burton and colleagues [1] demonstrated wide variability in performance on the Glasgow Face Memory Task (GFMT)–a task that was found to be moderately related to a simple test of face memory yet strongly related to an object matching test. Furthermore, Bindemann, Aveytisan, and Rakow [2] reported that participants’ performance on the same matching task can vary from one attempt to the next. In addition, studies adopting real-life paradigms have shown that instantaneous unfamiliar face matching is an error-prone task, even though there are no constraints on memory or decision time. For instance, Kemp, Towell and Pike [10] examined the accuracy of experienced cashiers when detecting fraud in a supermarket scenario. The authors reported that cashiers failed to reject a fraudulent card in 64% of trials where the bearer’s identity was similar (but not a match) to that on a photographic ID card. More strikingly, cashiers still made errors on 34% of trials where the image on a fraudulent ID card did not resemble the bearer at all. This error rate seems particularly high given that the participants in the study were aware of the experiment taking place and presumably were vigilant and motivated to perform the task well. A more recent study by White, Kemp, Jenkins, Matheson and Burton [11] investigated the performance of passport officers in comparison to lay persons on a task resembling a passport-matching scenario. The study reported (1) a 14% rate of acceptance of fraudulent IDs by passport officers, (2) that performance on a matching task was unrelated to the length of relevant occupational experience, and (3) the passport officers did not outperform lay participants. These findings are somewhat surprising given that poor performance on unfamiliar face matching tasks has previously been attributed to the novelty of the task. That is, people typically only process faces that are known to them, and are rarely required to assess the identity of unknown faces [4]. Because passport control officers perform these tasks on a daily basis and receive training aimed at enhancing their face matching skills, this explanation clearly cannot account for White et al.’s findings.

The findings reviewed above suggest that individuals who are particularly proficient at processing unfamiliar faces may be useful employees in occupations where excellent face matching skills are necessary, a solution previously suggested in the applied literature [1–3]. In the UK, the current practice is to employ trained facial image analysts to examine the similarity of two images, for instance when doubts arise in a courtroom. In support of this procedure, numerous recent studies have shown that trained experts are better at face matching than individuals without experience in facial image comparison [12–14]. Of particular interest is a report by White and colleagues [13] showing that forensic experts outperform student participants at face matching across three different tests. Interestingly, while these trained specialists were more accurate than students on all administered tasks, the same was not true in two out of three tests for another control group recruited amongst staff from the same organisation but without experience in assignments involving face matching. This finding casts some doubt on the appropriateness of student control groups used in studies investigating expertise. It is possible, that while experts are motivated to do well, students who typically have to collect a number of experimental credits to pass a course do not possess the same motivation and disengage their attention when faced with a difficult task involving face matching.

However, it is important to note that there is little evidence to suggest that typical perceivers can be successfully trained in facial image comparison, and it may be that the aforementioned experts are naturally better at unfamiliar face processing. Some studies have reported no training-related improvements at all [15–17], although recent investigations have noted minor enhancements with feedback [18] cf. [19] or when matching decisions are made in pairs [20].

Given the large individual differences that have been observed in face matching skills and the limited effectiveness of training programmes, an alternative is to initially select personnel based on their natural face recognition ability. Pertinently, recent work has identified people with superior face recognition skills: so called “super recognisers” (SRs) [21]. These individuals report extraordinary face recognition performance in everyday life, as confirmed by performance on a standardised laboratory test assessing memory of facial identity (the Cambridge Face Memory Test-Long Form, CFMT+) [21]. Further evidence suggests that at least some SRs also excel at more applied tasks involving face memory, such as recognising previously studied faces from moving video clips or matching faces in line-up arrays [3]. However, very little work has examined face perception skills in super recognition, and it is currently unknown whether these individuals also excel at simple instantaneous matching tasks resembling ID to person matching situations. Although some authors have investigated the performance of SRs on the Cambridge Face Perception Test [21,22], it should be noted that performance was only examined at the group rather than the individual level (where significant differences are impossible to detect due to the high control mean and large standard deviation associated with this task), and the test has little resemblance to critical real-world tasks that require accurate face matching skills.

Investigation of the face matching skills of SRs is an important theoretical issue given individuals at the other end of the face recognition spectrum (i.e. those with prosopagnosia: [23–25]) can present with or without impairments in face perception [26]. Such findings have aided the development of dominant models of face-processing (e.g. Bruce & Young [7]), by indicating that the face-processing pathway can be lesioned at different locations (i.e. at an early stage involving structural encoding or a later stage involving retrieval); yet the presumed hierarchical nature of the framework explains why the hallmark deficit in facial identity recognition presents even in the former group of individuals. Likewise, it follows that super recognition may result from relatively early enhancements affecting facial identity perception, or from later enhancements affecting memory for faces; and the underpinnings of the skills may vary between individuals. Examination of the face perception abilities of SRs will therefore have important theoretical implications by presenting a novel means to evaluate current theoretical frameworks, and also has real-world value by assessing the capabilities of these people (both in a group and at individual level) in tasks that are fundamental to national and international security. Indeed, the prosopagnosia literature raises the possibility that only some SRs will also excel as tasks involving face matching.

The current study addresses this issue by investigating whether seven SRs are better at matching faces (i.e. face perception) than opportunistically chosen samples of typical perceivers. First, participants completed the Glasgow Face Matching Test (GFMT) [1]–a task that has been extensively used in previous research [2,27,28]. However, because overall accuracy in the GFMT task is high even in typical perceivers, it is hard to detect significant differences in performance on an individual level. With the aim of exacerbating these disparities, we also used a more demanding face matching task, the Models Face Matching Test (MFMT, [20]). In this test, images are of models who undergo a change in appearance between images. Thus, this task examined whether any superior face matching skills of SRs are also evident when task demands are high, and efficient extraction of information is required in order to elicit an accurate response. Given that these tests resemble tasks that are typically performed by passport control and other security officers, this investigation is of particular interest to those who perform person-to-ID comparisons in an occupational setting.

## Method

### Ethics statement

All participants gave full written consent and the experiment was approved by the ethics committee of the Psychology department at Bournemouth University.

### Participants

Seven SRs (four male) participated in this task (see Table 1). All SRs contacted our laboratory following media coverage of super recognition. As in previously published work, their superior face recognition skills were confirmed using the CFMT+ and the published age-appropriate norms that are available for this test [3,21,22].

**Table 1:** Demographical information and CFMT+ scores for the SR participants used in this study and those described by Russell, Chatterjee, and Nakayama [22]. Standard deviations are in parentheses.

	Controls	Motivated Controls	Russell et al.’s SRs	The current study							
	(N = 20)	(N = 20)	(N = 6)	SRs(N = 7)	SR1	SR2	SR3	SR4	SR5	SR6	SR7
Age	25.2 *(5*.*6)*	24.2 (5.0)	40.7 *(9*.*9)*	25.0 *(5*.*4)*	27	29	20	27	33	20	19
Gender	10 F	10 F	-^1^	3 F	M	M	F	F	M	M	F
CFMT+	71.1 *(10*.*5)*	71.8 (12.7)	95.0 *(1*.*9)*	97.7 *(3*.*2)*	101	97	96	94	100	95	96

^1^Gender data was not available for Russell et al.’s [22] participants.

In addition, 20 typical perceivers were recruited from students and visitors at the University to act as controls. All control participants were also screened using the CFMT+, to exclude those meeting the criteria for either super recognition or prosopagnosia [29]. However, no exclusions were necessary. They were matched to the SR group according to age and gender (10 male). All participants had normal or corrected-to-normal vision, and participated on a voluntary basis or in exchange for course credits. Because of the concerns related to how well student participants are motivated to perform tasks in group studies, an additional gender- and age-matched group was recruited offering a financial incentive. Specifically, this group of 20 individuals were informed that they would receive reimbursement for their time in accordance with the departmental policy (£8 per hour of participation), but they would also gain an extra £1 for every 10% increase in accuracy above 50% (chance performance) on the face matching tasks. In sum, this group was aware that the better they perform the tasks, the more money they would get in addition to their statuary reimbursement. These participants are referred to as “motivated controls” in the remainder of this manuscript.

Importantly, all seven SR participants recruited for this study outperformed both control groups on the CFMT+, in both group and case-by-case analyses (see Table 1). The latter were performed using modified t-tests for single case comparisons [30] (all *p*s < .05). Ethical approval for the study was granted by Bournemouth University’s Ethics Committee.

### Materials and Procedure

#### Glasgow Face Matching Test (GFMT)

The original GFMT (long version) is comprised of 168 pairs of male and female faces: half contain faces of the same identity and half do not [1]. All images were 350 pixels in width (the images were standardised by width and their height varied naturally) and were displayed in greyscale at a resolution of 72ppi without noticeable jewellery or clothing, but hairstyle was visible. The 84 people used in the matched trials were also paired with a distractor on mismatched trials. The distractors were chosen based on their similarity to the target images, adopting a sorting procedure used by Bruce et al. [4]. All participants were tested individually using E—Prime software (Psychology Software Tools, Sharpsburgh, PA, USA) and a 22 inch LCD monitor displayed at a resolution of 1920 x 1080 pixels. Participants sat approximately 60cm from the screen, and made their responses using the *s* and *k* keys on a keyboard under no time constraints. Each individual saw all 168 pairs, and the trials were presented in a randomised order.

#### Models Face Matching Test (MFMT)

The MFMT is comprised of 120 pairs of male faces: 60 matched according to identity and 60 mismatched (for further stimuli details see Dowsett & Burton) [20]. All images measured 300 *(W)* x 420 *(H)* pixels and were displayed in colour to mimic natural settings when face matching would occur. The images did not contain any visible jewellery, but were not occluded of clothing and the hair was not cropped. As for the GFMT, similarity ratings for the mismatched trials were gathered using the method devised by Bruce et al.[4]. Each individual saw all 120 pairs of faces, with the two images matching on half of the trials. The trials were presented in a randomised order. The equipment, procedure and instructions were identical to those used in the GFMT.

### Statistical Analyses

For each task a 2 x 3 mixed factorial design was used, with a within—subjects factor of trial type (matched/mismatched) and a between—subjects factor of participant group (controls/motivated controls/SRs). The percentage of hits (correct responses in matched trials), misses (no—match decisions in matched trials), correct rejections (correct “mismatched” responses in mismatched trials), and false positive responses (match decisions in mismatched trials) were calculated for each participant.

While accuracy is a good indicator of the overall patterns of responses between the SR and control groups, additional analyses regarding sensitivity and response criterion permit a more in-depth understanding of the differences between them, and are useful for cross-study comparison [31]. Specifically, *d prime* (*d’*), a measure of sensitivity, was calculated by subtracting the *z* scores for false—positive (F) responses in the mismatched trials from *z* scores calculated from match responses (hits, H) in matched trials [*d’* = *z*(H)–*z*(FPs)] (see Table 2). Response bias (*criterion c*) was calculated as the negative average sum of *z* scores for the hits and false-positive response: *c* = -0.5[*z*(H) + *z*(FPs)] [32].

**Table 2:** Group accuracy descriptive statistics in GFMT. Standard deviations are in parentheses.

	Matched trials	Mismatched trials	Total Accuracy
	Hits (%)	Correct Rejection (%)	(%)
**SRs**	97.02 *(3*.*73)*	97.7 *(1*.*98)*	97.36 *(2*.*16)*
**Controls**	91.31 *(6*.*81)*	88.45 *(7*.*5)*	87.43 *(5*.*26)*
**Motivated controls**	96.20 *(3*.*62)*	84.55 *(10*.*93)*	87.85 *(5*.*45)*

## Results

### Glasgow Face Matching Test

Accuracy: A 2 x 3 mixed analysis of variance (ANOVA) was conducted on accuracy scores, with trial type as the within—participant factor (matched/mismatched) and group as the between-participant factor (SRs/controls/motivated controls). Means and SDs performance of all three groups re displayed in Table 2. There was a significant main effect of group, *F*(2, 44) = 6.12, *p* = .005, η_p_^2^ = .218 and a post hoc Tukey test revealed that SRs performed better than controls (*p* = .004) and motivated controls (*p* = .008), and that there were no overall differences in accuracy between the two control groups (*p* = .949). There was also a main effect of trial type whereby all participants were more accurate on matched trials *F*(1, 44) = 7.47, *p* = .009, η_p_^2^ = .145. These main effects were qualified by a significant interaction between participant group and trial type, *F*(2, 44) = 5.45, *p* = .008, η_p_^2^ = .198.

Follow-up analyses were conducted for matched and mismatched trials. For matched trials, a one-way ANOVA revealed significant differences between groups, *F*(2, 44) = 5.49, *p* = .007, η_p_^2^ = .200. Planned comparisons showed that SRs were more accurate than controls (*p* = .049) but not motivated controls (*p* = .932), who in turn were more accurate than the standard control participants (*p* = .004). There were also significant between-group differences in mismatched trials, *F*(2, 44) = 5.89, *p* = .005, η_p_^2^ = .211. SRs were marginally better than controls (*p* = .052), significantly better than motivated controls (*p* = .004), and there were no significant differences between both control groups (*p* = .344).

Signal detection analyses: A one-way ANOVA on *d* prime scores revealed significant differences between the three groups of participants, *F*(2, 44) = 10.91, *p* = .< .001, η_p_^2^ = .332 (see Table 3). Follow-up analyses revealed that SRs were better at discriminating pairs of faces than controls (*p* < .001) and motivated controls (*p* = .001), and there were no differences in sensitivity between the two control groups (*p* = .456). Furthermore, a one-way ANOVA on criterion *c* scores indicated differences between the three groups of participants, *F*(2, 44) = 5.42, *p* = .008, η_p_^2^ = .198. There were no differences in response bias between the SRs and controls (*p* = .691), but the motivated control group was more prone to elicit a “yes” response than either SRs (*p* = .023) and, interestingly, controls *(p* = .028). On an individual level, modified *t*-tests for single case comparisons were performed [33] on the *d* prime and criterion *c* data for each of the SRs in comparison to the control groups. This method was specifically developed for single case studies in neuropsychology where, instead of a large set of normative data, individuals are compared to small samples (N < 50) of control participants. Three SR participants (SR1, SR2 and SR 5) showed significantly higher sensitivity than controls on the matching task and, importantly, none of the SRs displayed a different response bias to control participants. In contrast to the motivated controls, the same three SR participants were better at discriminating simultaneously presented faces and one was more prone to reject image pairs as mismatched (SR3).

**Table 3:** Individual case analyses of sensitivity of SRs in GFMT, using modified t-tests for single-case comparisons [30].

	Mean *(SD)*	Single-case comparisons						
		SR1	SR2	SR3	SR4	SR5	SR6	SR7
***d prime***								
SRs	4.23 *(0*.*74)*	5.03	5.03	3.50	4.32	4.78	3.65	3.34
Controls	2.82 (0.73)	.**008**	.**008**	.374	.059	.**016**	.281	.495
Motivated Controls	3.08 (0.63)	.**007**	.**007**	.523	.069	.**016**	.388	.692
***criterion c***								
SRs	0.02 *(0*.*27)*	0	0	0.51	-0.36	0.13	0.16	0
Controls	-0.11 (0.32)	.741	.741	.074	.455	.473	.420	.741
Controls Motivated	-0.39 (0.38)	.329	.329	.**032**	.939	.197	.174	.329

### Models Face Matching Test

#### Accuracy

A 2 x 3 mixed analysis of variance (ANOVA) was conducted on accuracy scores, with trial type as the within—participant factor (matched/mismatched) and group as the between-participant factor (SRs/motivated controls/controls). Means and SDs performance of all three groups are displayed in Table 4. There was a significant main effect of group, *F*(2.44) = 16.4, *p* < .001, η_p_^2^ = .427, and post hoc Tukey tests revealed that SRs performed better than controls (*p* < .001) and motivated controls (*p* = .001), and the difference between the two control groups was marginally significant (*p* = .059). There was also a main effect of trial type whereby all participants were more accurate on mismatched trials, *F*(1, 44) = 6.45, *p* = .015, η_p_^2^ = .128. The interaction between these two factors was non-significant *F*(2, 44) = 1.99, *p* = .049, η_p_^2^ = .083.

**Table 4:** Group accuracy descriptive statistics in MFMT Standard deviations are in parentheses.

	Matched trials	Mismatched trials	Total Accuracy
	Hits (%)	Correct Rejection (%)	(%)
**SRs**	71.90 *(14*.*92)*	88.19 *(5*.*31)*	82.5 *(6*.*29)*
**Controls**	63.33 *(17*.*66)*	65.58 *(13*.*74)*	64.46 *(7*.*46)*
**Motivated controls**	67.65 *(12*.*24)*	71.95 *(9*.*79)*	69.80 *(7*.*19)*

#### Signal detection analyses

A one-way ANOVA on *d* prime scores revealed significant differences between the three groups of participants, *F*(2, 44) = 22.79, *p* = .< .001, η_p_^2^ = .509 (see Table 4). Follow-up analyses indicated that SRs were better at discriminating pairs of faces than controls (p < .001) and motivated controls (p < .001), and there were no differences in sensitivity between the two control groups (*p* = .128). Furthermore, a one-way ANOVA on criterion *c* scores indicated differences between the three groups of participants, *F*(2, 44) = 4.4, *p* = .018, η_p_^2^ = .167. SRs were more likely to report a mismatch than both controls (*p* = .019) and motivated controls (p = .023), and there were no differences in bias between the two control groups (*p* = .986). On an individual level, modified *t*-tests for single case comparisons were performed on the *d* prime and criterion *c* data for each of the SRs in comparison to the two control groups (see Table 5). All but one SR (SR3) were better at the MFMT than the control group, and four SRs (SR1, SR2, SR4, SR6) were better at discriminating simultaneously presented faces in comparison to the motivated control group. Two SRs (SR2 and SR7) were also more biased towards classifying image pairs as mismatched than both the control and motivated control groups.

**Table 5:** Individual case analyses of sensitivity of SRs in MFMT using modified t-tests for single-case comparisons [30].

	Mean *(SD)*	Single-case comparisons						
		SR1	SR2	SR3	SR4	SR5	SR6	SR7
***d prime***								
SRs	2.23 *(0*.*46)*	3.1	2.38	1.68	2.35	2.11	2.22	1.83
Controls	0.82 (0.46)	<.**001**	.**004**	.083	.**004**	.**013**	.**007**	.**045**
Motivated Controls	1.12 (0.5)	.**001**	.**023**	.288	.**027**	.068	.**045**	.182
***criterion c***								
SRs	0.48 *(0*.*4)*	0.58	0.94	0.54	-0.21	0.33	0.27	0.92
Controls	0.03 (0.41)	.203	.**042**	.237	.573	.481	.573	.**046**
Controls Motivated	0.05 (0.3)	.101	.**009**	.127	.401	.373	.483	.**011**

### GFMT versus MFMT performance

**Fig 1:**
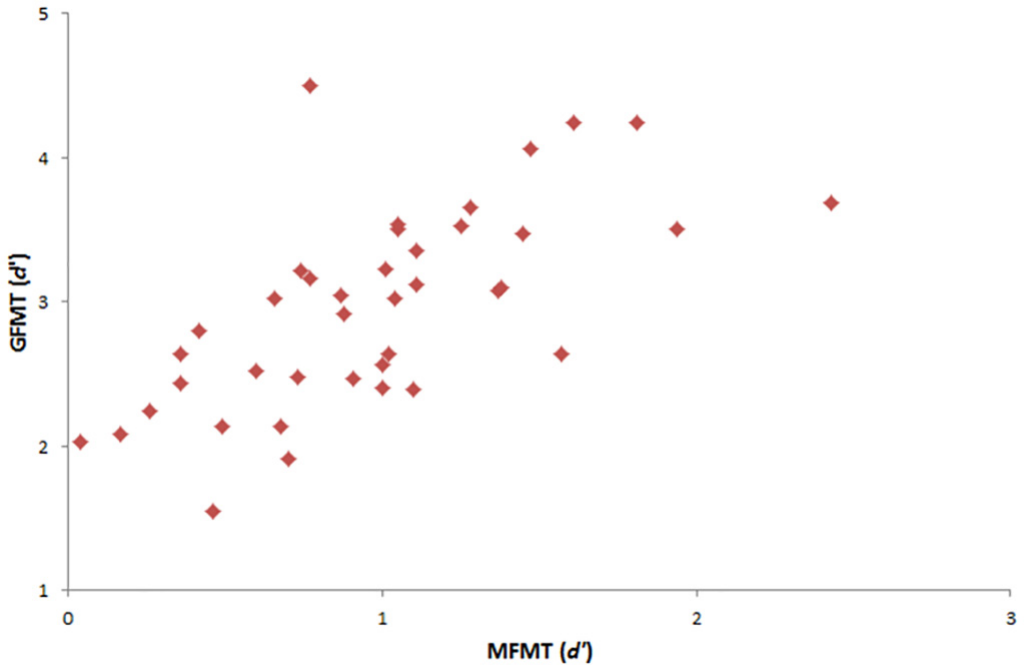
Correlation between GFMT and MFMT performance for all control participants.

Because there were no demographic or baseline face recognition ability differences (*p*s < .05) between participants in both control groups, performance (*d* prime) of all control participants on the GFMT task was correlated with performance on the new, more challenging MFMT test to examine the stability of matching judgments over the two tasks. Analyses revealed that performance on the two tests was strongly correlated, *N* = 40, *Spearman’s rho* = .719, *p* < .001 (see Fig 1).

## General Discussion

In this investigation, the face matching performance of seven SRs was compared to that of two groups of typical perceivers on the GFMT and the more-demanding MFMT. As predicted, group (and most case-by-case) analyses indicated that SRs outperformed controls on both tasks. The SRs also displayed a more conservative response bias (i.e. they were more likely to reject an image pair as mismatched) on the MFMT task at the group and, in two cases, individual level. There was also a strong positive correlation between the GFMT and MFMT scores of all control participants which indicated some stability in face matching performance across the two tasks. These findings have implications for both theory and practice.

In terms of theory, the work presented here is a further testimony to the considerable variation in human face matching ability that has been reported in previous work [1,2,34], i.e. while some participants were highly accurate on the matching tasks reported here, others were less so. This range of performance was particularly apparent in the more demanding MFMT (the minimum accuracy was 51% and 53% in control and motivated groups, respectively, while the top performer, SR1, was accurate on 91% of the trials). What is more, the selective enhancement in face matching ability in the SR group provides some insight into the memory-perception dissociation in face processing [1,34]. Specifically, while all the SRs showed enhanced face memory on a standardised laboratory task (the CFMT+), not all of these individuals outperformed controls on the matching tasks. That is, not all of the SRs were “super matchers”. This finding converges with previous work by Burton and colleagues [1] who reported small correlations between a simple face memory and the GFMT, but a strong correlation between the GFMT and an object memory task. The authors concluded that unfamiliar faces are processed in a similar way to objects. While the two experiments presented here do not have scope to support the latter proposition, they offer some evidence that memory for faces and face perception are not overlapping constructs. Specifically, the relative dissociation between face memory (measured by the CFMT+) and face matching (assessed using the GFMT and MFMT) in the SR group provides some ground for critique of the Bruce and Young’s [7] sequential model of face recognition. The model posits that face perception precedes face recognition and the latter cannot be achieved without the initial perceptual input. Although it is possible that intact, but not enhanced, face perception may be enough for face memory to be enhanced at a later stage, the mechanism behind such augmentation is unclear. The inverse of this effect has been previously observed in the developmental prosopagnosia literature, where all individuals have impaired face memory, but some present with face perception ability on par with that of typical perceivers [24,26]. A recent study by Bobak and colleagues [3] further speaks to this issue: seven SRs excelled as a group and, mostly on individual level, at face matching in a line-up paradigm, but only one showed exceptional performance on an individual level in a demanding face memory task with a delayed recognition phase (they outperformed the controls in the memory task in group analyses, however).

Without well-defined evidence for direct forward-feeding of perceptual input, the results of this study are better explained by the Interaction Activation and Competition Model (IAC, [35]) where pools of units can be activated in parallel. It is possible that this simultaneous activation of perception and memory occurs more strongly in some SRs than others, resulting in enhanced face matching concomitant with excellent recognition memory for faces. In a bid to further clarify this issue, future studies should aim to examine long-term memory for faces in the SR population.

Furthermore, it is also possible that “super matchers” may exist who do not necessarily have superior memory for faces. Indeed, in the current study the range of performance between the control groups and the SRs was somewhat overlapping, i.e. the lowest performing SRs in both the GFMT and MFMT were less accurate than the highest performing control participants. A similar pattern of findings was also reported in our recent work [3], where control participants with “average” face memory displayed a broad range of abilities in their face matching performance. From a theoretical and practical perspective, future work examining individual differences should therefore screen participants on different aspects of face-processing, with a particular emphasis on dissociating face perception and face recognition. One possible way of investigating this phenomenon would be via the use of eye-tracking technology. There is convincing evidence to suggest that eye movements are pivotal in face learning [36,37] and it is possible that eye-movement behaviour also underpins skilful face matching. Future studies may wish to investigate this phenomenon in more depth using tasks that are applicable to situations resembling ID-to-person checks.

Further, an alternative explanation for super recognition is that the skill is simply underpinned by more generalized processes, such as those supporting memory, perception or even global precedence. If this is the case, screening for individuals with these abilities may be fruitful, particularly if they are more susceptible to face training than those with more average generalized abilities. Pertinently, a recent study with twin pairs identified that 61% variance in face recognition ability is heritable and independent from other domains such as general memory, intelligence and object processing [38]. It is thus possible that the exceptional performance of SRs is contingent on their genetic make-up. Hence, future work should also examine the face-specificity and heritability of super recognition, for both practical and theoretical benefits.

However, it is important to note that results from two matching tasks described in this report converge with another recent study with SR participants, where Bobak and colleagues ([3], Experiment1) reported a somewhat inconsistent performance amongst seven SRs on another applied task of face perception, the one-in-ten test [4]. Pertinently, while all the SRs in Bobak et al.’s study showed enhanced performance on the CFMT+ and outperformed controls in the two experimental tasks on group-based analyses, only four SRs displayed significantly higher performance on a case-by-case basis. The scores of the three remaining participants, although all at least 1SD above the mean of the control group, did not reach statistical significance on a case-by-case basis. The current study presents a broadly similar pattern of findings. While these differences were not pronounced for the SR and control groups on the GFMT, the SRs always outperformed control participants by more than 1SD on in the more challenging MFMT task. The lack of differences on the former test may be accounted for by the high accuracy in the control groups. As it is hard to detect differences on individual level when there is little room for improvement in experimental paradigms, future studies should take heed of our findings and employ carefully constructed paradigms with sufficient accuracy level to allow such enhancements to be detected.

The individual variability reported in this study raises some important questions about conclusions drawn on the performance of groups of experts. In their recent studies, White and colleagues [13,14] argued that forensic analysts are better than control groups (student and “motivated” employees of governmental agencies) at applied matching tasks. However, while the individual data are available, these studies merely presented group statistics and it is unclear whether these group differences may have been driven by particularly exceptional performance of some experts while the accuracy of others may have been on par with the control groups. It is recommended that future studies examining face matching accuracy in applied settings report case-by-case analyses in addition to group performance, taking special care to ensure that the anonymity of participants working in national security settings is protected. Furthermore, the results reported here suggest that motivation to perform well is not a key issue in face matching accuracy. Specifically, monetary payment with an explicit incentive to do well had little effect on the performance of two demographically similar control participant groups. In addition, while the overall accuracy and sensitivity remained at the same level amongst all control participants, motivated controls were more somewhat likely to elicit a “yes” response on all trials. Given that this bias was not replicated in both face matching tasks, it is unclear how this phenomenon may be interpreted. Nonetheless, this finding contributes to the body of literature showing limited effects of enhancement techniques and motivation on face processing ability [10,15,19].

In terms of practice in the national security settings, given the typically high error rates and limited success of face matching training regimes [16,17,19] cf. [20], this study lends further support to the notion that selection of individuals with excellent face matching skills could be a useful recruitment strategy for occupations where ID-to-person matching is a pivotal part of the job. Indeed, SRs identified by self-report and a standardised test of face recognition memory, the CFMT+, were also mostly found to be excellent face matchers. This advantage was particularly evident in the difficult MFMT task, where all but one SR outperformed control participants. It is of note that SRs not only outperformed typical perceivers on both matched and mismatched trials, but they also displayed a more conservative response bias on the more difficult MFMT. This cautious approach to accepting an identity match is a particularly valuable skill in national security settings, and provides further evidence of SRs’ utility in these occupations. Accepting a fraudulent ID (akin to eliciting a “yes” response on a mismatched trial) is a potentially costly mistake to make during ID checks and, as such, examination of response bias may also be a valuable procedure during recruitment. While such predisposition may be slowing the processing of applicants on national frontiers, in the wake of calls to tighten national security, this potential deceleration of processing speed is a rather minor expense in comparison to potential threat from individuals carrying fraudulent IDs.

Furthermore, White and colleagues [11] reported that performance on the GFMT predicts photo ID-to-person matching performance, in that individuals who are apt at laboratory-based matching tasks are also high performers in real world ID-to-person comparison tasks, and vice versa. However, White et al. produced ID cards from photographs taken only a few days before the testing session. In real life, passports are typically issued for a period of 10 years—a time in which facial appearance can significantly change. These circumstances heighten the task demands faced by passport officers [6]. As such, the MFMT used in the current study may be an even better indicator of face matching ability in real—life settings. The stimuli were taken from a database of models where some images are original photographs provided by the models themselves, and some come from later photo shoots where the model has changed appearance (e.g. their hairstyle). These circumstances mimic real-world scenarios to a greater extent than previous laboratory-based face matching tasks. Future studies should examine the predictive value of the MFMT on real-life passport security checks, to establish whether it would be a useful recruitment tool.

## Conclusion

In sum, the results presented here show that (1) SRs as a group (and mostly on an individual level) are significantly better than controls at applied tasks of face matching, (2) face matching performance is stable across different tasks, and (3) face matching ability is independent of participants’ motivational level. Recent research has repeatedly shown that there are large individual differences in unfamiliar face recognition and facial identity perception [21,39], including individuals at one extreme who are particularly skilled at face-processing (SRs) and those at the other extreme who have severe difficulties in facial identity recognition (developmental prosopagnosia). A lack of effective training techniques makes border control agencies vulnerable to costly mistakes in identity judgement, and these agencies would benefit from having SRs in their ranks. While not infallible, SRs are clearly better at face matching, especially when task demands are high, and they would be an asset for national security agencies.

## Supporting Information

S1 Dataset Accuracy and d prime for GFMT and MFMT. (SAV)
